# Optimal control design of turbo spin‐echo sequences with applications to parallel‐transmit systems

**DOI:** 10.1002/mrm.26084

**Published:** 2016-01-22

**Authors:** Alessandro Sbrizzi, Hans Hoogduin, Joseph V. Hajnal, Cornelis A. T. van den Berg, Peter R. Luijten, Shaihan J. Malik

**Affiliations:** ^1^ Imaging DivisionUniversity Medical Center UtrechtUtrechtThe Netherlands; ^2^ Biomedical Engineering Department, Division of Imaging SciencesKings College LondonEngland, UK

**Keywords:** optimal control, extended phase graph, parallel transmit radiofrequency, direct signal control, fast spin‐echo, turbo spin‐echo

## Abstract

**Purpose:**

The design of turbo spin‐echo sequences is modeled as a dynamic optimization problem which includes the case of inhomogeneous transmit radiofrequency fields. This problem is efficiently solved by optimal control techniques making it possible to design patient‐specific sequences online.

**Theory and Methods:**

The extended phase graph formalism is employed to model the signal evolution. The design problem is cast as an optimal control problem and an efficient numerical procedure for its solution is given. The numerical and experimental tests address standard multiecho sequences and pTx configurations.

**Results:**

Standard, analytically derived flip angle trains are recovered by the numerical optimal control approach. New sequences are designed where constraints on radiofrequency total and peak power are included. In the case of parallel transmit application, the method is able to calculate the optimal echo train for two‐dimensional and three‐dimensional turbo spin echo sequences in the order of 10 s with a single central processing unit (CPU) implementation. The image contrast is maintained through the whole field of view despite inhomogeneities of the radiofrequency fields.

**Conclusion:**

The optimal control design sheds new light on the sequence design process and makes it possible to design sequences in an online, patient‐specific fashion. Magn Reson Med 77:361–373, 2017. © 2016 The Authors Magnetic Resonance in Medicine published by Wiley Periodicals, Inc. on behalf of International Society for Magnetic Resonance in Medicine

## INTRODUCTION

Turbo spin‐echo (TSE) sequences 1 based on the Carr–Purcell–Meiboom–Gill (CPMG) condition 2, 3 are extensively used in current clinical MRI exams. Originally intended as a rapid succession of $180^{o}$ refocusing pulses 4, the spin‐echo technique has been applied with trains of lower refocusing angles 5, considerably reducing the power deposition in the tissue and also allowing for much longer echo trains.

Previous work has addressed the design of optimal, low refocusing angle trains 6, 7, 8. In particular, Ref. 6 shows how in some cases the angles can be analytically derived for a number of multiecho sequence types such as hyperechoes 9 and transition between pseudo steady states (TRAPS) 10. Other work has focused on the optimization of the tip angle trains, usually in a heuristic fashion 11, 12, 13.

An important extension of the standard sequence design is presented in 14, 15, where the parallel transmit (pTx) system characteristics are taken into account for patient specific calculations. At high and ultra high field MRI, the Larmor frequency is such that interference and penetration effects for radiofrequency (RF) waves occur in the body. As a consequence, the RF transmit field is no longer homogeneous, leading to inconsistent contrast and signal loss over the field of view. pTx systems consist of independent and simultaneously superimposed RF fields. Many researchers have proposed methods for using the new degrees of freedom available from pTx 16, 17, 18. In the context of improving signal homogeneity, these may be divided into those which aim to improve uniformity of the RF field directly like RF shimming 19; those which aim to improve uniformity of properties of individual RF pulses using multidimensional RF pulses [e.g., “spokes” 20, “*k*_T_‐points” 21, 22, 23 or “SPINS” 24]; or finally those which seek to directly consider the resulting signals 14, 15. The latter methods have been proposed for TSE sequences since they do not require multidimensional pulses that may increase the achievable interecho spacings. Instead, signal evolution over the entire pulse sequence is considered. This approach differs from other recent work on TSE sequences 23 which must take care to design pulses whose phases are matched to obey the CPMG conditions as Massire et al. mentioned in the discussion of 23. By directly considering the resulting echo amplitudes, this is implicitly accounted for.

Previous work on signal control has used simple numerical optimization to compute optimized settings leading to long computation times. This is a weakness since these calculations must be carried out while the subject is in the scanner. In particular, use of gradient based optimization with finite difference approximations is computationally costly. Whilst later work has employed a nongradient based approach 15, the convergence of such methods remains slow since the structure of the minimization problem is not exploited.

In this work, a fundamentally different approach is taken, in which we analyze the structure of the extended phase graph (EPG) algorithm and provide insights in the modeling of the sequence design process. The EPG is seen as a discrete‐time dynamical system. The design problem is cast as the minimization of a smooth function under smooth equality and/or inequality constraints. As all functions involved in the model are differentiable, the resulting numerical optimal control problem can be solved by efficient derivative‐based methods. As a consequence, the computation time to determine the optimal sequence settings is such that online clinical implementation becomes feasible.

The exact derivatives of all functions playing a role in the model are presented below. For the functions which implicitly depend on the design parameters, the adjoint states method (ASM) is used. This algorithmic differentiation method makes it possible to efficiently calculate the exact derivatives of a dynamical process 25. Note that ASM was previously applied to large‐tip angle RF pulses design for pTx systems in 26.

We will give several examples of optimal control EPG‐based design. First, we will show how standard, analytically derived sequences are recovered by the algorithm. Among these cases, the derivation of the CPMG condition through numerical optimization is included. Next, we will design new sequences and finally we will apply the same framework to patient specific, pTx configurations. In the latter case, application of optimal control with ASM allows online, patient specific computations as the total computation time is in the order of 10 s on a standard desktop PC with a single CPU implementation. Finally, the optimized pTx TSE sequence will be experimentally tested on a phantom and on a volunteer's head with an eight‐channel transmit system at 7T MRI.

The new optimal control framework is a generalized and flexible approach to sequence design and can efficiently calculate sequences on a patient‐specific basis; it is useful as a support for MR scientists and as an online tool for high‐field pTx MRI exams.

This code is made available online at https://github.com/mriphysics/optimal-control-EPG.

## THEORY

### The Extended Phase Graph and Its Extension to pTx Configurations

We will start by giving a basic description of the EPG. More detailed information can be found in the review article of Weigel 27.

The EPG describes the evolution of the signal in terms of configuration states ${\left(F_{k}^{+},F_{k}^{-},Z_{k}\right)}^{T}$. For sequences with equidistant timing (fixed *T*_R_ and *T*_E_), they are defined as the Fourier coefficients of the complex magnetization states ${\left(M_{+},M_{-},M_{z}\right)}^{T}$:

(1) $$\begin{array}{c} F_{k}^{+}\propto \int_{\Theta }M_{+}(\theta )e^{-\iota k\theta }d\theta \\ F_{k}^{-}\propto \int_{\Theta }M_{-}(\theta )e^{-\iota k\theta }d\theta \\ Z_{k}\propto \int_{\Theta }M_{z}(\theta )e^{-\iota k\theta }d\theta \end{array}$$

with:

(2) $$\begin{array}{c} M_{+}(\theta )=M_{x}(\theta )+\iota M_{y}(\theta )=\sum_{k=-\infty }^{\infty }F_{k}^{+}e^{\iota k\theta }\\ M_{-}(\theta )=M_{x}(\theta )-\iota M_{y}(\theta )=\sum_{k=-\infty }^{\infty }F_{k}^{-}e^{\iota k\theta }\\ M_{z}(\theta )=\sum_{k=-\infty }^{\infty }Z_{k}e^{\iota k\theta }. \end{array}$$

In the above equations, θ∈[0,2π] quantifies the dephasing due to off‐resonance caused by applied gradient fields and $\iota =\sqrt{-1}$.

The echo value is given by the two *F*_0_ states. Note the complex conjugate relationship: $F_{k}^{+}={\left(F_{-k}^{-}\right)}^{*}$.

The Bloch equation dictates the dynamics of ${\left(M_{+},M_{-},M_{z}\right)}^{T}$, which can be decomposed into rotation, relaxation and dephasing effects. In the EPG formalism, these three phenomena are given respectively by: the rotation operator, R, the relaxation operator E, and the shift operator S, which are defined as:

(3) $$R(\alpha ,\phi )=\left(\begin{array}{ccc} \cos^{2}(\alpha /2) & \exp(2\iota \phi )\sin^{2}(\alpha /2) & -\iota \exp(\iota \phi )\sin(\alpha )\\ \exp(-2\iota \phi )\sin^{2}(\alpha /2) & \cos^{2}(\alpha /2) & \iota \exp(-\iota \phi )\sin(\alpha )\\ -\iota /2\exp(-\iota \phi )\sin(\alpha ) & \iota /2\exp(\iota \phi )\sin(\alpha ) & \cos(\alpha ) \end{array}\right)$$

(4) $$E=\left(\begin{array}{ccc} \exp(-\tau /T_{2}) & 0 & 0\\ 0 & \exp(-\tau /T_{2}) & 0\\ 0 & 0 & \exp(-\tau /T_{1}) \end{array}\right)$$

(5) $$S:\;F_{k}\mapsto F_{k+1}\;\text{and}\;Z_{k}\mapsto Z_{k}.$$

In the previous equations, *α*, ϕ, *T*_1_, *T*_2_, and *τ* represent, respectively, the tip angle amplitude, the corresponding phase, the longitudinal and transverse relaxation rates, and the echo spacing. In the case of variable refocusing angles, we write *α_n_* and $\phi_{n}$ where the subscript *n* indicates the pulse number.

The EPG calculation is obtained by recursively applying the three operators to the configurations states ${\left(F_{k}^{+},F_{k}^{-},Z_{k}\right)}^{T}$.

### Spatially Resolved EPG and pTx Configurations

In the case of a *L*‐channel pTx system, the *effective* amplitude *α_n_* and phase $\phi_{n}$ are derived from the sum of all complex RF pulse weights multiplied by the $B_{1}^{+}$ sensitivity of the corresponding transmit coil:

(6) $$\alpha_{n}=\left|\sum_{\ell =1}^{L}B_{\ell }*(x_{n,\ell }+\iota y_{n,\ell })\right|\;\text{and}\;\phi_{n}=∠\left\{\sum_{\ell =1}^{L}B_{\ell }*(x_{n,\ell }+\iota y_{n,\ell })\right\}$$

where $B_{\ell }$ denotes the complex transmit field of the ℓ‐th channel and $x_{n,\ell }$ and $y_{n,\ell }$ represent, respectively, the real and imaginary part of the flip‐angle applied to the ℓ‐th transmit channel at the *n*‐th pulse. Note that $B_{\ell }$ is dimensionless. For implementation reasons (see the Appendix), it is convenient to use the real/imaginary part representation for the complex RF weights of the pTx system. Needless to say, the (α,ϕ) and (*x*, *y*) representations are interchangeable. RF pulse amplitudes are given in terms of *nominal* flip angle: that is, the input to the system. The flip angle effectively achieved by the RF system is the nominal value multiplied by the $B_{1}^{+}<1$ spatial distribution of the corresponding channel.

The transmit fields are usually spatially dependent, a fact that is reflected in the spatial response of the echo train. Therefore, the EPG needs to be evaluated in each voxel of the region of interest, leading to the spatially resolved version of the EPG 14.

### Sequence Design as an Optimization Problem

The EPG can be formulated as a discrete‐time dynamical process:

(7) $$\{\begin{array}{ccc} f_{n+1} & = & P_{n}f_{n}\\ f_{0} & = & b \end{array}$$

where *f_n_* is the 3(K+1)×1 vector of all concatenated states at the *n*‐th time point:

(8) $$f_{n}={\left(F_{0}^{+},F_{0}^{-},Z_{0},F_{1}^{+},F_{1}^{-},Z_{1},\ldots ,F_{K-1}^{+},F_{K-1}^{-},Z_{K-1},F_{K}^{+},F_{K}^{-},Z_{K}\right)}_{n}^{T}.$$

*P_n_* represents the *transition* matrix, which depends on the sequence and tissue parameters $\alpha_{n},\phi_{n},\tau ,T_{1}$ and *T*_2_. *K* is the maximum number of configuration states.

For example, $P_{n}=R(\alpha_{n},\phi_{n})E(\tau ,T_{1},T_{2})S$ represents dephasing followed by decay and refocusing. The matrices *S*, *E* and *R* are constructed by concatenating the refocusing, decay and rotation matrices from the previous section into block‐diagonal matrices (see the Appendix for more implementation details). For fixed $\tau ,T_{1}$ and *T*_2_, the free parameters are $\alpha =(\alpha_{0},\ldots ,\alpha_{N-1})$ and $\phi =(\phi_{0},\ldots ,\phi_{N-1})$. Finally, *b* represents the initial state (typically all zero components but the one corresponding to $Z_{0}=1$).

The time index *n* runs from 0 to *N* and represents the echo numbers, in particular: *n* = 0 is the initial or equilibrium state, *n* = 1 is the state right after the excitation and *n* = 2 is the first echo. For the largest coefficient, *K*, in Eq. (8) we have *K* = *N*. Note that Malik et al. show in 15 that for large *n*, the effect of the large coefficients *k* is small. In this work, we have chosen to truncate the series in *k* such that K=min{N,25}. This allows for a considerable acceleration in the computations.

The signal at echo‐time *n* – 1 is given by the first and the second components of *f_n_*, which are the conjugate of one another. In this work we will thus consider only the second component.

The sequence design will be cast as a minimization problem of the standard form:

(9) $$\{\begin{array}{ll} \text{Minimize} & s(\alpha ,\phi )\\ s.t. & p_{i}(\alpha ,\phi )=0\;(i=1,\ldots ,I)\\ & q_{j}(\alpha ,\phi )\leq 0\;(j=1,\ldots ,J) \end{array}$$

where *s*, *p,* and *q* represent, respectively, the objective, the equality, and inequality constraints functions. *I* and *J* denote, respectively, the number of equality and inequality constraints. The functions *s*, *p,* and *q* are required to be differentiable.

For example, to design a sequence which maximizes the signal intensity over the whole echo train, we can set:

(10) $$s=-\frac{1}{2}\sum_{n=2}^{N}f_{n}^{H}C_{n}f_{n}.$$

In this equation, *C_n_* is a diagonal weighting matrix which selects only the signal component of *f_n_* (*f_n_* contains all states), that is:

$$C_{n}(j,j)=c_{n}\neq 0\;\text{if}\;j=2\;\text{and}\;C_{n}(j,j)=0\;\text{otherwise}.$$

For example, setting *c_n_* = 0 for n≤M means that the signal from the first *M* echoes is not taken into account. As default, *c_n_* = 1, but it can be convenient to assign larger weights to the central k‐space echoes. We can easily implement this by setting, for instance, *c_n_* = 2 for these particular k‐space indices.

As another example, suppose we want to design a sequence whose signal closely follows a predefined target response, *t*, and the total RF power has to be minimized. In this case we set

(11) $$s(\alpha ,\phi )=\frac{1}{2}\sum_{n=0}^{N-1}\alpha_{n}^{2}$$

(12) $$q_{1}(\alpha ,\phi )=\left[\frac{1}{2}\sum_{n=2}^{N}{\left(f_{n}-t_{n}\right)}^{H}C_{n}(f_{n}-t_{n})\right]-\sigma^{2}$$

where $\sigma^{2}$ denotes the maximum allowed deviation from the desired target. We only specify the echo component of *t_n_* and the others are zero.

Upper bounds for the tip angle amplitude can be set in the following way:

(13) $$q_{j}(\alpha ,\phi )=\alpha_{j}-\alpha_{\max},\;(j=0,\ldots ,N-1)$$

where $\alpha_{\max}$ denotes the maximum values allowed.

In case of pTx configurations, the objective and/or constraint functions are evaluated over a set of *R* spatial positions. Making use of the real/imaginary representation, the design problem is easily extended.

The building block functions for the optimal control design are schematically presented in Table 1. Clearly, the spatially resolved version of the design problem can be applied also to single channel transmit configurations.

**Table 1:** Building Block Functions for the Optimal Control Problem

	Standard setup	pTx configuration
Error function	$\frac{1}{2}\sum_{n=2}^{N}{\left(f_{n}-t_{n}\right)}^{H}C_{n}(f_{n}-t_{n})$	$\frac{1}{2}\sum_{r=1}^{R}\sum_{n=2}^{N}{\left(f_{n,r}-t_{n,r}\right)}^{H}C_{n}(f_{n,r}-t_{n,r})$
Signal amplitude	$-\frac{1}{2}\sum_{n=2}^{N}f_{n}^{H}C_{n}f_{n}$	$-\frac{1}{2}\sum_{r=1}^{R}\sum_{n=2}^{N}f_{n,r}^{H}C_{n}f_{n,r}$
Total RF power	$-\frac{1}{2}\sum_{n=0}^{N-1}\alpha_{n}^{2}$	$-\frac{1}{2}\sum_{n=0}^{N-1}\sum_{\ell =1}^{L}x_{n,\ell }^{2}+y_{n,\ell }^{2}$
Peak RF power	$\alpha_{n}-\alpha_{\max}$for n=0,…,N−1	$x_{n,\ell }^{2}+y_{n,\ell }^{2}-\alpha_{\max}^{2}$for n=0,…,N−1 and ℓ=1,…,L

The subscript r=1,…,R indicates the spatial position.

### Calculating the Exact Derivatives. The Adjoint States Method

To efficiently solve the optimal control problem given by Eq. (9), the derivative of all functions involved with respect to the design parameters is needed. Calculating the derivatives of the functions which explicitly depend only on the parameters is rather straightforward. For example, we have:

(14) $$\begin{array}{c} s=\frac{1}{2}\sum_{n=0}^{N-1}\alpha^{2}\Rightarrow \frac{\partial s}{\partial \alpha_{j}}=\alpha_{j}\\ q=\alpha_{j}-\alpha_{\max}\Rightarrow \frac{\partial q}{\partial \alpha_{j}}=1\\ q=x_{n,\ell }^{2}+y_{n,\ell }^{2}-\alpha_{\max}^{2}\Rightarrow \frac{\partial q}{\partial x_{n,\ell }}=2x_{n,\ell }\;\text{and}\;\frac{\partial q}{\partial y_{n,\ell }}=2y_{n,\ell } \end{array}$$

Conversely, the derivatives of the functions which implicitly depend on the design parameters can not be directly determined. Examples thereof are given in Table 1.

In these cases, the derivative could be approximated by finite differences schemes. For example:

$$\frac{\partial s}{\partial \alpha_{j}}\approx \frac{s(\alpha_{0},\alpha_{1},\ldots ,\alpha_{j}+\delta ,\alpha_{j+1}\ldots )-s(\alpha_{0},\alpha_{1},\ldots ,\alpha_{j},\alpha_{j+1}\ldots )}{\delta }$$

for a small value of *δ*. The advantage of this solution is the simplicity of its implementation. However, each derivative requires an EPG simulation. When dealing with a large amount of parameters and voxels (for the spatially resolved EPG), the repeated evaluation of *s* becomes prohibitively long.

An extremely efficient alternative to solve this problem is offered by the ASM. ASM is a very powerful tool, extensively used in numerical solutions of optimal control problems. It consists of a forward/backward simulation approach to compute *exact* derivatives. We will show how ASM can be applied to the problem of finding $\frac{\partial s}{\partial \alpha_{j}}$ by a backward recurrence strategy.

Suppose we want to calculate the derivatives of

$$s=\frac{1}{2}\sum_{n=2}^{N}{\left(f_{n}-t_{n}\right)}^{H}C_{n}(f_{n}-t_{n}).$$

First of all, note that the derivative of *s* with respect to the last parameter, $\alpha_{N-1}$, is given by:

$$\frac{\partial s}{\partial \alpha_{N-1}}=\frac{\partial s}{\partial f_{N}}\frac{\partial f_{N}}{\partial \alpha_{N-1}}={\left(f_{N}-t_{N}\right)}^{H}C_{N}\frac{\partial f_{N}}{\partial \alpha_{N-1}}={\left(f_{N}-t_{N}\right)}^{H}C_{N}\frac{\partial P_{N-1}}{\partial \alpha_{N-1}}f_{N-1}$$

where we used the fact that $f_{N}=P_{N-1}f_{N-1}$. Note that the derivatives of $P_{N-1}$ are analytically defined.

Defining the (N−1)‐th adjoint state, $\lambda_{N-1}$:

$$\lambda_{N-1}\equiv {\left(f_{N}-t_{N}\right)}^{H}C_{n}\;\Rightarrow \;\frac{\partial s}{\partial \alpha_{N-1}}=\lambda_{N-1}\frac{\partial P_{N-1}}{\partial \alpha_{N-1}}f_{N-1}.$$

The next step consists of calculating $\frac{\partial s}{\partial \alpha_{N-2}}$:

(15) $$\frac{\partial s}{\partial \alpha_{N-2}}=\frac{\partial }{\partial \alpha_{N-2}}\left[{\left(f_{N-1}-t_{N-1}\right)}^{H}C_{N-1}(f_{N-1}-t_{N-1})+{\left(f_{N}-t_{N}\right)}^{H}C_{N}(f_{N}-t_{N})\right]={\left(f_{N-1}-t_{N-1}\right)}^{H}C_{N-1}\frac{\partial P_{N-2}}{\partial \alpha_{N-2}}f_{N-2}+{\left(f_{N}-t_{N}\right)}^{H}C_{N}P_{N-1}\frac{\partial P_{N-2}}{\partial \alpha_{N-2}}f_{N-2}$$

where we used the fact that $f_{N}=P_{N-1}P_{N-2}f_{N-2}$. Rearranging the right‐hand side terms:

(16) $$\begin{array}{c} \frac{\partial s}{\partial \alpha_{N-2}}=\left[{\left(f_{N-1}-t_{N-1}\right)}^{H}C_{N-1}+{\left(f_{N}-t_{N}\right)}^{H}C_{N}P_{N-1}\right]\\ \frac{\partial P_{N-2}}{\partial \alpha_{N-2}}f_{N-2}=\left[{\left(f_{N-1}-t_{N-1}\right)}^{H}C_{N-1}+\lambda_{N-1}P_{N-1}\right]\frac{\partial P_{N-2}}{\partial \alpha_{N-2}}f_{N-2}. \end{array}$$

Defining the (N−2)‐th adjoint states:

$$\lambda_{N-2}\equiv {\left(f_{N-1}-t_{N-1}\right)}^{H}C_{N-1}+\lambda_{N-1}P_{N-1}\Rightarrow \;\frac{\partial s}{\partial \alpha_{N-2}}=\lambda_{N-2}\frac{\partial P_{N-2}}{\partial \alpha_{N-1}}f_{N-2}.$$

Continuing this process, we obtain the backward recurrence relation for the adjoint states:

(17) $$\{\begin{array}{ccc} \lambda_{N-1} & = & {\left(f_{N}-t_{N}\right)}^{H}C_{N}\\ \lambda_{N-2} & = & \lambda_{N-1}P_{N-1}+{\left(f_{N-1}-t_{N-1}\right)}^{H}C_{N-1}\\ & \vdots & \\ \lambda_{n} & = & \lambda_{n+1}P_{n+1}+{\left(f_{n+1}-t_{n+1}\right)}^{H}C_{n+1}\\ & \vdots & \\ \lambda_{0} & = & \lambda_{1}P_{1}+{\left(f_{1}-t_{1}\right)}^{H}C_{1}. \end{array}$$

Once the adjoint states are found, the derivatives can be easily calculated:

(18) $$\frac{\partial s}{\partial \alpha_{n}}=\lambda_{n}\frac{\partial P_{n}}{\partial \alpha_{n}}f_{n}$$

and, in an analogous way, we observe that

(19) $$\frac{\partial s}{\partial \phi_{n}}=\lambda_{n}\frac{\partial P_{n}}{\partial \phi_{n}}f_{n},\;\;\frac{\partial s}{\partial x_{n,\ell }}=\lambda_{n}\frac{\partial P_{n}}{\partial x_{n,\ell }}f_{n}\;\text{and}\;\frac{\partial s}{\partial y_{n,\ell }}=\lambda_{n}\frac{\partial P_{n}}{\partial y_{n,\ell }}f_{n}.$$

The derivatives of the transition matrix *P_n_* in Eqs. (18) and (19) are analytically obtained in the Appendix.

### Piecewise Constant Sequence Parameters and Reduction of Degrees of Freedom

In practice, it is useful to reduce the degrees of freedom of the sequence design by constraining *α_n_* and $\phi_{n}$ to be constant over a certain time interval. The consequence is that the design variables are reduced in number, improving convergence performance of the algorithm and making it possible for the scanner interface to deal with fewer input values.

In particular, suppose that we set:

$$(\alpha_{n},\phi_{n})=(a_{j},b_{j}),\;\forall n\in Q_{j}$$

where $Q_{j}=\{\eta_{0},\eta_{1},\ldots \}$ is a set of indices indicating the *j*‐th range. For example, setting $Q_{0}=\{0\},Q_{1}=\{1\},Q_{2}=\{2,3,4\},Q_{3}=\{5,6\}$ means that

(20) $$\{\begin{array}{ccc} \left(a_{0},b_{0}\right) & = & \left(\alpha_{0},\phi_{0}\right)\\ \left(a_{1},b_{1}\right) & = & \left(\alpha_{1},\phi_{1}\right)\\ \left(a_{2},b_{2}\right) & = & \left(\alpha_{2},\phi_{2})=(\alpha_{3},\phi_{3})=(\alpha_{4},\phi_{4}\right)\\ \left(a_{3},b_{3}\right) & = & \left(\alpha_{5},\phi_{5})=(\alpha_{6},\phi_{6}\right) \end{array}$$

The design parameters for the flip angle amplitudes are then reduced from 14 to 8. In matrix form:

(21) $$\left(\begin{array}{c} \alpha_{0}\\ \alpha_{1}\\ \alpha_{2}\\ \alpha_{3}\\ \alpha_{4}\\ \alpha_{5}\\ \alpha_{6} \end{array}\right)=\left(\begin{array}{cccc} 1 & 0 & 0 & 0\\ 0 & 1 & 0 & 0\\ 0 & 0 & 1 & 0\\ 0 & 0 & 1 & 0\\ 0 & 0 & 1 & 0\\ 0 & 0 & 0 & 1\\ 0 & 0 & 0 & 1 \end{array}\right)\left(\begin{array}{c} a_{0}\\ a_{1}\\ a_{2}\\ a_{3} \end{array}\right)$$

which can be more compactly be written as

α=Qa.

Analogously, we can write ϕ=Qb. The derivatives with respect to the new variables are easily calculated from the derivatives with respect to *α* by application of the chain rule:

$$\frac{\partial s}{\partial a}=\frac{\partial s}{\partial \alpha }\frac{\partial \alpha }{\partial a}=\frac{\partial s}{\partial \alpha }Q\;\text{and}\;\frac{\partial s}{\partial b}=\frac{\partial s}{\partial \phi }\frac{\partial \phi }{\partial b}=\frac{\partial s}{\partial \phi }Q.$$

The same method can be applied when the flip angles are expressed in terms of real and imaginary components.

### Computational Complexity of the ASM for EPG

The main computational burden in the optimal control method lays in: (a) the forward recursion scheme for the configuration states; (b) the backward recursion scheme for the adjoints states given by Eq. (17); and (c) the two loops to calculate the products in Eqs. (18) and (19). Note that the last two loops can be combined into a single one, which is twice as costly in computational terms. Each of these four loops requires a similar amount of floating point operations as an EPG simulation. It follows that the computational burden for each iteration approximates four EPG calculations.

As shown in 25, the ASM becomes very attractive when the number of parameters is large. To give an example, in the case of *N* = 50 excitations train, with both amplitude and phase as variable gives 100 derivatives to be calculated for each iteration step of the minimization algorithm. The finite difference approximating method would require $2\times N_{p}+1$ EPG simulations, where *N*_p_ denotes the possibly reduced number of design parameters (see also the example from the previous paragraph, where *N*_p_ = 4). If all degrees of freedom are maintained, that is *N* = *N*_p_, then the EPG simulation has to be repeated 101 times. The computational cost for ASM still approximates four EPG simulations.

For a pTx configuration, the signal response is calculated for several voxels in the region of interest, increasing the simulation time of the EPG. For example, in 14 approximately 75 spatial points were used, increasing the number of EPG calculations by a factor of 75. The scale of the problem increases dramatically and the minimization problem needs to be solved online. In the pTx case, the design variables are the real and imaginary component of the pulse for each *n* and each transmit channel. For an eight‐channel pTx system, the total number of parameters is $2\times 8\times N_{p}$. The time reduction factor of ASM with respect to finite difference is thus $(2\times 8\times N_{p})/4=4N_{p}$. The application of ASM is fundamental to compute the optimal sequence parameters online.

Details about the practical implementation are given in the Appendix.

## METHODS

In this section, we test the optimal control design approach. We run the minimization problem for some sequence designs whose solution is analytically known and compare the outcomes. Afterwards, we show how to easily extend the requirements on the sequence by adding constraints. Finally, we address the pTx, spatially resolved EPG configurations.

In all tests, the minimization problem is solved by the built‐in Matlab implementation of the interior‐point method (function fmincon) with user‐defined gradients of the objective and constraint functions (see also the Appendix). The Hessian is approximated by the Broyden–Fletcher–Goldfarb–Shanno method 28. The spatially resolved EPG simulator is implemented in C++ by making use of the linear algebra library Armadillo 29 on a Linux PC with Intel Xeon CPU, 3.50 GHz. The Matlab and C++ functions are interfaced by means of file streams. The code does not make use of parallelization thus only one CPU core is employed.

### Test 1. Maximizing the Signal on a Standard TSE

In the first test, we wish to design a sequence which maximizes the signal, that is:

(22) $$\begin{array}{cc} \text{Minimize} & -\frac{1}{2}\sum_{n=2}^{N}f_{n}^{H}C_{n}f_{n}. \end{array}$$

We choose $\left(T_{1},T_{2})=(1000,150\right)$ ms, *N* = 60. As a starting guess, we set $\alpha_{0}=90^{o}$ and $\alpha_{n}=60^{o}$ for n=1,…,N. The starting phases, $\phi_{n}$, obey the CPMG condition, that is $\phi_{0}=0^{o}$ (excitation pulse) and $\phi_{n}=90^{o}$ for n≥1 (refocusing pulses). We expect to recover the standard turbo‐spin‐echo sequence given by $\alpha =(90^{o},180^{o},180^{o},\ldots ,180^{o})$.

### Test 2. Constant Signal Intensity with Minimum RF Power

For the second test, we wish to calculate the minimum total power tip angle train for a sequence which returns constant signal intensity given by *I*_c_. The analytical solution, omitting relaxation, is given by 6:

$$\alpha_{n}=2\arctan\left(\frac{1}{2}(I_{c}-F_{-2}^{e}(n-1))/Z_{1}^{e}(n-1)\right).$$

The design problem can be cast as:

(23) $$\{\begin{array}{ll} \text{Minimize} & \frac{1}{2}\sum_{n=1}^{N-1}\alpha_{n}^{2}\\ s.t. & \frac{1}{2}\sum_{n=2}^{N}{\left(f_{n}-t_{n}\right)}^{H}C_{n}(f_{n}-t_{n})-\sigma^{2}\leq 0 \end{array}$$

where $t_{n}(2)=I_{c}$ and *σ* a small positive number which controls the accuracy of the obtained signal intensity.

We solve the problem for three values of *I*_c_, namely: $I_{c}\in \{0.3,0.6,0.9\}$ and we set $\sigma =10^{-3}$. We omit relaxation by setting the decay operator E=I. As a starting guess, we set $\alpha_{0}=90^{o},\;\alpha_{n}=18^{o}$ for n=1,…,N−1 and $\phi_{n}=0$.

### Test 3. Exploiting Flexibility. Peak RF‐Constrained TSE and Recovery of the CPMG Condition

The numerical optimal control approach has the advantage of being very flexible. For instance, assume that we wish to design a maximum signal sequence with refocusing angles smaller than $60^{o}$ and we are interested only in the signal after the fifth echo. This can be easily implemented as:

$$\{\begin{array}{ll} \text{Minimize} & -\frac{1}{2}\sum_{n=7}^{N}f_{n}^{H}C_{n}f_{n}\\ s.t. & \alpha_{n}\leq 60^{o}\;n=1,\ldots ,N-1 \end{array}$$

We set $\left(T_{1},T_{2})=(1000,150\right)$ ms and the echo‐train length is *N* = 60. The starting candidate is $\alpha_{0}=90^{o},\;\alpha_{n}=60^{o}$ for n=1,…,N and $\phi_{n}=0$.

This test is divided in two parts. In the first part, we require the refocusing pulses to be constant (see the paragraph on piecewise constant sequence parameters in the Theory section). This means that *α_n_* and $\phi_{n}$ for n≥1 will be represented by a unique numerical value. The excitation pulse is independent from the refocusing pulses. The aim of this numerical experiment is to investigate whether the CPMG condition is recovered by the optimal control approach.

In the second part of this test, we leave complete freedom to each refocusing pulse to vary in time.

### Test 4: Eight‐Channel pTx System at 7 Tesla. 3D ROI Optimization on a Phantom

In the next two tests we investigate the sequence design for pTx systems. We consider an eight‐channels transmit system at 7.0 Tesla. A transceive headcoil (Nova Medical, Wilmington, DE) and a 7T scanner (Philips, Best, NL) are used. In the first scanner test, a spherical phantom of 10 cm diameter is used. It contains the following compounds: acetate, ethanol, phosphoric acid, and arquad solution. The approximated *T*_1_ and *T*_2_ values are 500 ms and 400 ms, respectively. The relative $B_{1}^{+}$ maps for the eight channels are derived from low flip‐angle gradient‐echo images, as described in 30. In addition, an absolute $B_{1}^{+}$ mapping measurement is performed with the DREAM method 31 and is used to recover quantitative information about the flip angle; the resulting maps are plotted in Figure 5a. We design optimal amplitude and phase settings for a three‐dimensional‐turbo‐spin‐echo scan (3D‐TSE, also known as SPACE, or VISTA), TSE factor = 113. The sequence should be able to compensate for the RF inhomogeneities in such a way that the signal follows the dynamics of a predefined ideal echo train over the whole region of interest (ROI). By the word “ideal” we indicate a pulse train which is designed under the assumption of a perfectly homogeneous transmit field. In particular, we look at pseudosteady‐state sequences as derived in 32.

Constraints on the RF total and peak power are also required. The resulting problem in terms of the real, *x*, and imaginary, *y*, parts of the pulse settings is:

$$\{\begin{array}{lll} \text{Minimize} & \frac{1}{2}\sum_{r=1}^{R}\sum_{n=2}^{N}{\left(f_{n,r}-t_{n,r}\right)}^{H}C_{n}(f_{n,r}-t_{n,r}) & \left(\text{signal}‐\text{homogenization}\right)\\ s.t. & x_{n,\ell }^{2}+y_{n,\ell }^{2}\leq \alpha_{\max}^{2}\;n=0,\ldots ,N-1\;\ell =1,\ldots ,L & \left(\text{RF}\;\text{peak}\;\text{constraint}\right)\\ & \frac{1}{2}\sum_{n=0}^{N-1}\sum_{\ell =1}^{L}x_{n,\ell }^{2}+y_{n,\ell }^{2}\leq \Pi_{\max} & \left(\text{total}\;\text{power}\;\text{constraint}\right) \end{array}$$

where $\Pi_{\max}$ denotes the maximum allowed total power. The target signal $t_{n,r}$ is given by the EPG response of the echo train given by 32:

$$\alpha^{\text{standard}}=(90^{o},151^{o},90^{o},67^{o},60^{o},\ldots ,60^{o}).$$

The value $60^{o}$ is repeated 48 times to yield a total of 51 refocusing pulses (plus the excitation pulse). As the magnetization reaches the steady states during the first 51 pulses, there is no need to design also the remaining part of the sequence. The last value of *α* is repeated until the conclusion of the echo train.

**Figure 1:**
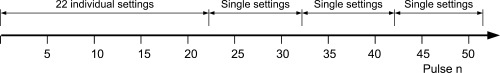
Diagram indicating the distribution of the optimization settings for test 4. The first 22 pulses are driven individually. The remaining 30 pulses are subdivided in three groups of equal length and each pulse group is represented by a single parameter. Note that the amplitude and phase for each of the eight channels are independently driven, resulting into (22+3)×8×2=400 optimization parameters.

To reduce the degrees of freedom, we force $\left(x_{n,\ell },y_{n,\ell }\right)$ to be constant in the three intervals 23≤n≤32, 33≤n≤42_,_ and 43≤n≤52. See also Figure 1 for the corresponding diagram. The number of design parameters is then reduced to 25×8×2=400 and the transmit maps are undersampled into a 3D tetrahedral lattice 15 to yield a total of *R* = 91 spatial locations.

We set $\alpha_{\max}=270^{o}$ and $\Pi_{\max}$ as twice the total power obtained by the nonoptimized sequence, $\alpha^{\text{standard}}$. This choice is empirically defined so as to remain within specific absorption rate constraints dictated by the power monitoring unit of the scanner. The starting guess is $\alpha^{\text{standard}}$ with $0^{o}$ and $90^{o}$ phase, respectively, for the excitation and refocusing pulses (CPMG condition). The optimal control algorithm is halted after 50 iterations.

The other sequence parameters are: $\left(T_{E},T_{R})=(188,3200\right)$ ms; echo‐spacing: 2.9 ms; echo‐train duration: 374 ms; resolution: 1 mm^3^; total scan duration: 10 min and 11 s.

### Test 5: In Vivo Scan with Eight‐Channel pTx System at 7 Tesla. 2D ROI Optimization

In the final test, we address the design of a two‐dimensional (2D) TSE sequence for a volunteer's brain scan. The $B_{1}^{+}$ maps for the eight channels are measured in the same way as in the previous test 30, 31 and they are displayed in Figure 7a. Constraints on peak and total RF power are also required. We obtain the same design problem as in the previous test.

The weights on the echoes (i.e., the nonzero entry of *C_n_*) are all equal to one except for the central k‐space echo, where we set it equal to 2.

The target signal intensity is given by the ideal (no $B_{1}^{+}$ inhomogeneity) EPG signal response of the echo train (TSE factor = 9):

$$\alpha^{\text{standard}}=(105^{o},174^{o},145^{o},140^{o},140^{o},140^{o},140^{o},140^{o},140^{o},140^{o}).$$

The 2D $B_{1}^{+}$ maps are undersampled by a factor 4 in both directions and only voxels corresponding to the cerebral region are taken into account. This step is automatically performed by the Brain Extraction Tool software 33. The resulting total number of voxels for the design problem is *R* = 104. The (*T*_1_, *T*_2_) reference values are set equal to the approximate relaxation times of White matter, that is 1500 ms and 100 ms, respectively 34. As for Test 4, the optimal control algorithm is halted after 50 iterations and the starting guess is $\alpha^{\text{standard}}$ with $90^{o}$ phase off‐set between the excitation and refocusing pulses.

The other sequence parameters are: $\left(T_{E},T_{R})=(60,3500\right)$ ms; echo‐spacing: 12 ms; echo‐train duration: 108 ms; resolution: 0.3 mm^2^; total scan duration: 7 min and 45 s.

We will compare the proposed method with the standard sequence run in circularly polarized mode.

## RESULTS

### Test 1. Maximizing the Signal on a Standard TSE

**Figure 2:**
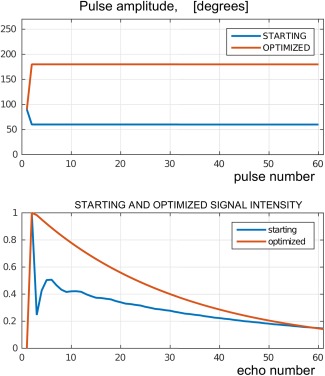
Test 1. Pulse amplitude (top) and signal intensity (bottom) for signal maximization without constraints on the RF. The phases for both pulse‐trains obey the CPMG condition. Note that the obtained train is the standard $90^{o}/180^{o}$ turbo spin echo.

The starting and optimized (α,ϕ) trains with the corresponding EPG signal responses are shown in Figure 2. As expected, the optimal control design recovers the standard $90^{o}/180^{o}$ spin‐echo sequence.

### Test 2. Constant Signal Intensity with Minimum RF Power

**Figure 3:**
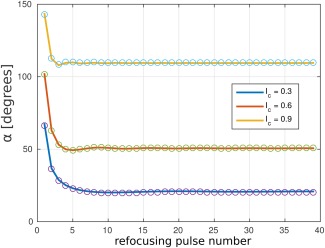
Test 2. The refocusing angles for constant signal intensities *I*_c_ obtained analytically (circles) and by optimal control design (continuous line). The two sets coincide.

The obtained refocusing angles for the constant signal intensity calculations are shown in Figure 3. Note that the analytical and algorithmic values coincide. We point out that the amplitudes are as shown in Figure 3 and the phases are unchanged from the CPMG phases.

### Test 3. Exploiting Flexibility. Peak RF Constrained TSE and Recovery of the CPMG Condition

**Figure 4:**
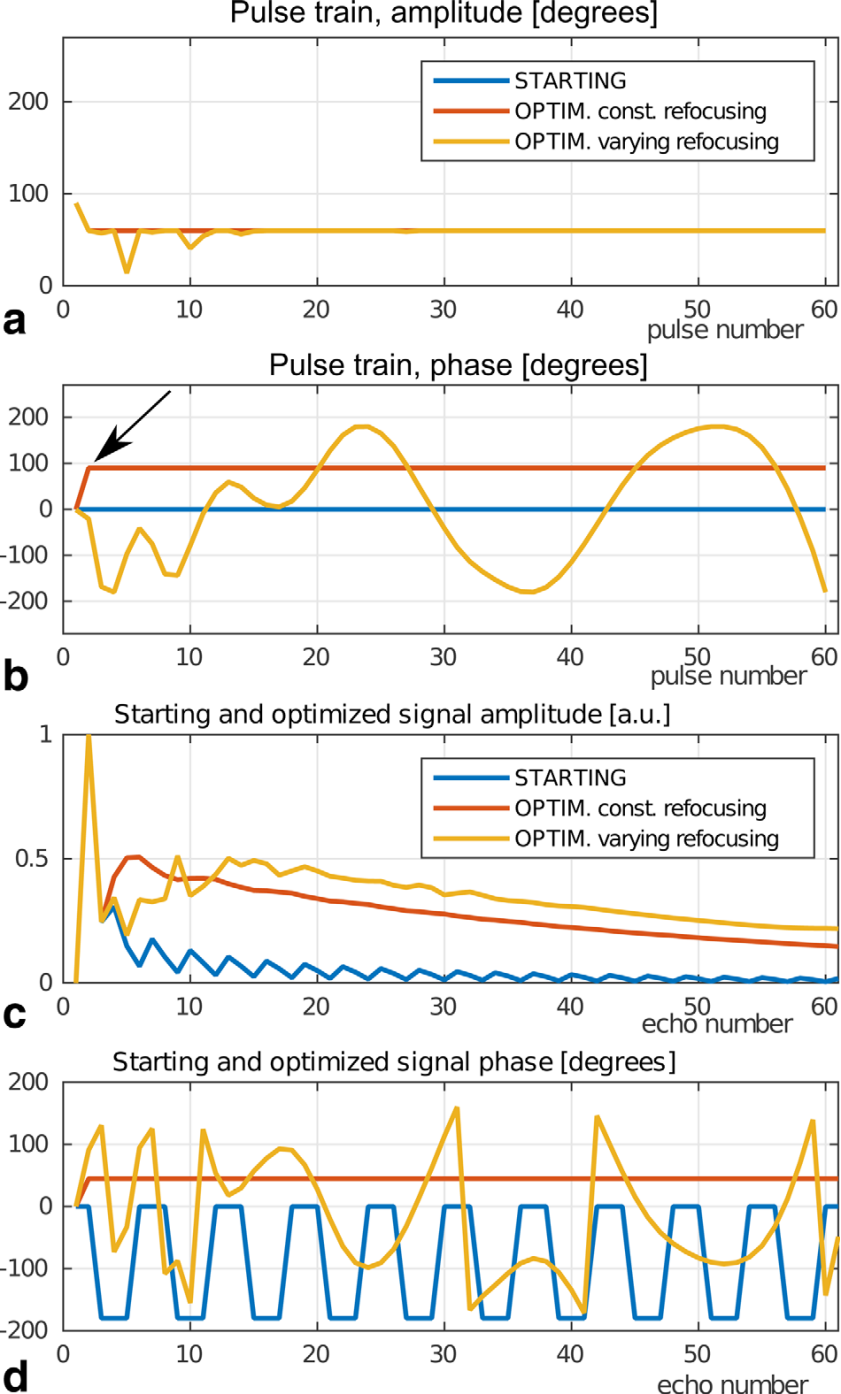
Test 3. Maximization of the signal when the refocusing angles are constrained by $60^{o}$. Constant and time‐varying refocusing angles are investigated. **a**: Amplitude (*α*) and (**b)** phase ( ϕ) modulation of the pulses. In part (a), the amplitude settings for the starting and the optimal constant refocusing angle setups coincide. Note the recovered CPMG condition in the optimal, constant refocusing angles setup. The excitation and refocusing angles have a $90^{o}$ phase off‐set (see arrow). **c**: Signal amplitude and (**d)** phase for each echo obtained from the three pulse trains. Note the increased signal intensity for the time‐varying with respect to the constant refocusing angle trains. The price to be paid is an additional phase modulation in the refocusing pulses, which is reflected in the phase of the signal.

Setting a maximum of $60^{o}$ on the refocusing angle, we obtain the pulse trains as shown in Figure 4.

For the constant refocusing angle setup, the optimized pulse train has the maximum allowed amplitude, that is, $60^{o}$ refocusing angle and a $90^{o}$ phase off‐set between the excitation and refocusing pulses (see the arrow in Fig. 4b). This is equivalent to the CPMG condition, which is thus recovered by the algorithm.

For the varying refocusing angle setup, the signal amplitude is the largest. Note in the latter case, the phase modulation of the optimized train. The consequence is an increase of the signal intensity with respect to the sequence where no phase modulation is present. The phase modulation given by the time‐varying refocusing pulses has to be accounted for prior to image reconstruction.

### Test 4: Eight‐Channel pTx System at 7 Tesla. 3D ROI Optimization on a Phantom

**Figure 5:**
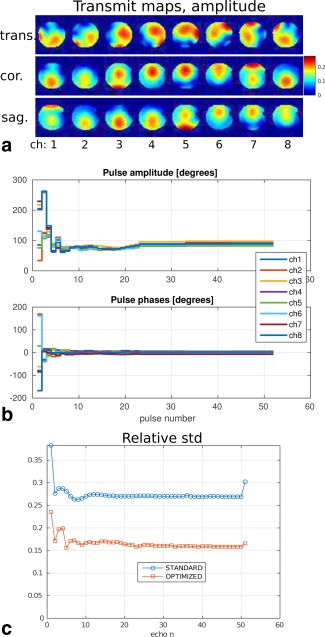
Test 4. (**a)** Amplitude‐transmit maps in the phantom for the eight‐channel system at 7T. Central transverse, coronal and sagittal slices are shown. **b**: Optimized amplitude and phases for the 3D TSE 113‐echo train (first 52 pulses are shown). The phase values correspond to the off‐set with respect to the standard (circularly polarized) excitation mode. **c**: Relative standard deviation of the signal intensity over the ROI for the two echo trains.

**Figure 6:**
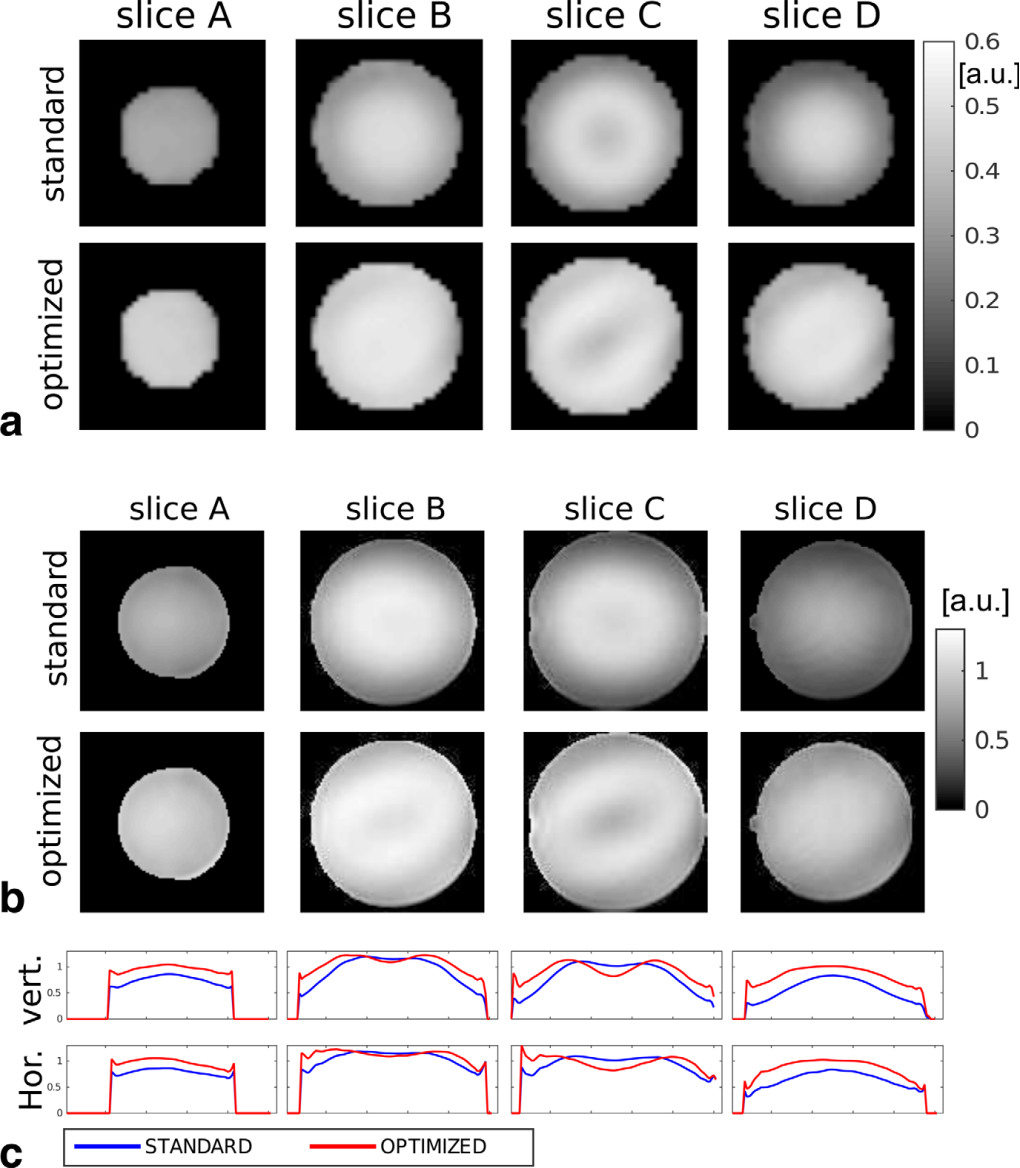
Test 4. Phantom 3D experiment. **a**: Simulated signal intensity for the 3D phantom experiment. Images correspond to four transverse slices separated by 20 mm. **b**: MR images for the 4 transverse slices. The images are scaled by the receive sensitivity profile. **c**: Profiles of the MR images taken along the central vertical and horizontal lines.

The computation time for the 8ch pTx 3D‐TSE sequence is about 45 s. The optimal amplitude and phase trains are shown in Figure 5b. The standard deviations of the signal intensities over the whole ROI divided by the mean value for each echo are plotted in Figure 5c. Figure 6a shows the simulated signal intensities for the central k‐space echo in four different transverse slices. The MR images obtained with this setup are shown in Figure 6b. These images are divided by the receive sensitivity profile and cropped to include only the phantom. In Figure 6c, the central vertical and horizontal image profiles are displayed. Note the improved homogeneity of the signal. Comparing Fig. 6a,b, we see that the predicted excitation patterns from the EPG simulations closely resemble the scanner images.

### Test 5: Eight‐Channel pTx System at 7 Tesla. 2D ROI Optimization, In Vivo Scanner Experiment

**Figure 7:**
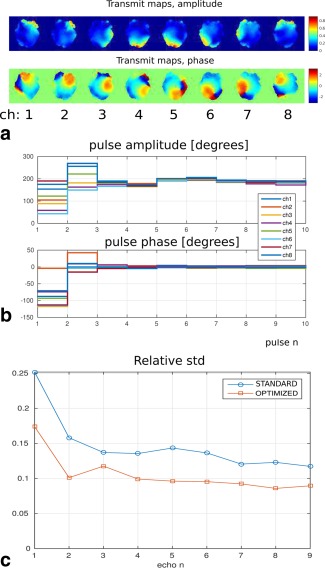
Test 5. (**a)** Amplitude and phase transmit maps in the volunteer's head for the 8 channel system at 7T. **b**: Optimized angles for the 2D TSE nine‐echo train. **c**: Relative standard deviation of the signal intensity for the two echo trains.

The computation time for the eight‐channel pTx 2D‐TSE sequence is about 8 s. The optimal amplitude and phase trains are shown in Figure 7b.

**Figure 8:**
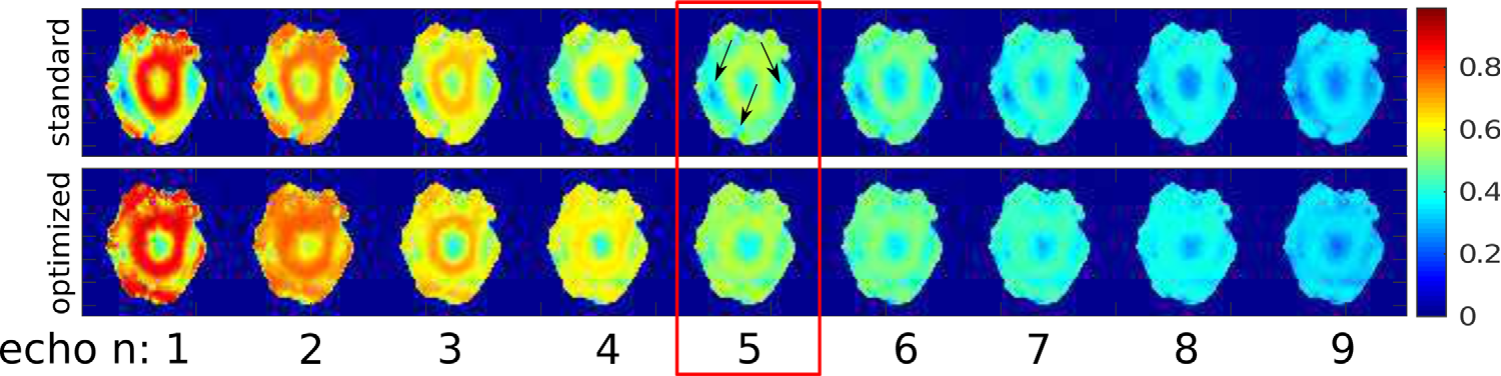
Test 5. Simulated echo amplitudes in the volunteer's head for the eight‐channel system at 7T. The echo corresponding to the central k‐space line is indicated by the red box. Top: standard quadrature excitation. Bottom: signal obtained with the optimized pTx settings. Note the improved homogeneity in the temporal regions (see arrows).

The relative standard deviations of the signal intensities over all brain voxels for each echo are plotted in Figure 7c. Note the improved homogenization level. The simulated signal amplitudes are shown in Figure 8. Echo number 5 corresponds to the central k‐space line acquisition (indicated by the box). The excitation fidelity is improved by the optimal control echo train.

**Figure 9:**
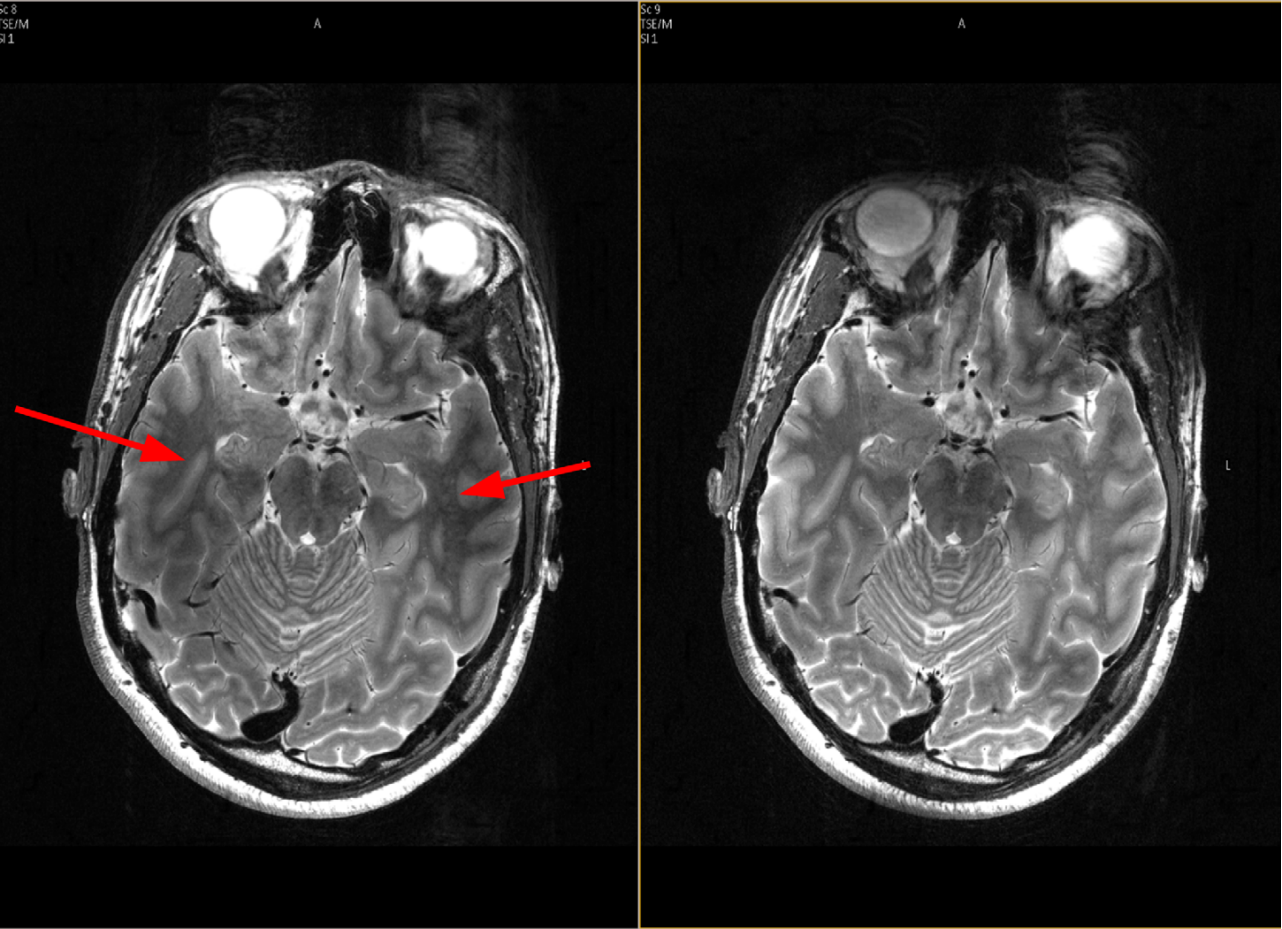
Test 5. In vivo MR experiment. Left: Image obtained by the standard 2D TSE sequence. The temporal lobes suffer of inhomogeneous contrast and signal loss (see arrows). Right: Image obtained with the Optimal Control pTx train. As predicted from the simulations, the contrast is maintained in the temporal lobes.

The in vivo images are shown in Figure 9. The contrast is more homogeneous over the whole brain and the signal is recovered also in the temporal regions (see arrows).

## DISCUSSION

We have presented a generalized and efficient numerical approach to the design of optimal pulse amplitudes and phases in fast spin‐echo sequences. The design problem is modeled as optimal control of differentiable functions. The resulting optimization procedure can thus be solved by efficient derivative‐based algorithms such as, for instance, the interior‐points method. Convergence to the known analytical solution is obtained for the simple cases where an analytic solution is available. Furthermore, the CPMG condition is shown to be recovered in test 3. This fact illustrates the robustness and validity of the presented approach. Additionally, we have shown how the flexibility of the algorithm can be exploited to design new sequences, revealing new insights in the field of sequence optimization. In particular, the sequence derived in test 3, where the refocusing angles are constrained by $60^{o}$, shows how a specific phase modulation can considerably increase the signal intensity. We believe that many more examples can be found where the interplay between varying amplitudes and phases lead to better sequences.

In the case of pTx systems at high fields MRI, the online optimization of the pulse train becomes necessary since the sensitivity profile of the RF transmit array is exam‐dependent. The numerical optimal control approach can work in a clinical environment thanks to the application of the ASM. This powerful algorithmic differentiation technique allows for time reduction factors in the order of 100 with respect to brute‐force finite differencing. The resulting computation time for a 3D TSE sequence and eight‐channel pTx system is in the order of 10 s, with a standard PC, single thread. The design problem can be easily solved by making use of multithreading implementation. Since the spatially resolved EPG and adjoint states simulations are voxel‐dependent, the calculations can be carried out simultaneously for different groups of voxels assigning each group to a separate thread. The acceleration factor in the computing time should be close to the number of threads used. As a result, the actual computing time for the most demanding sequences could be further reduced to few seconds. Furthermore, the derivatives obtained by ASM are exact, which means that the method is more robust to experimental imperfections and numerical errors.

The flexibility and speed of the proposed approach is exploited to homogenize the spatial response of the spin‐echo over the whole field of view in an eight‐channel pTx system at 7T. The results show a reduced standard deviation of the signal intensity for all echoes. The obtained amplitude and phase trains are limited by peak and total RF power but they still produce noticeable improvement in the in vivo image contrast as illustrated by the scanner experiments. The design problem could explicitly be formulated to maintain a uniform signal difference between tissue types. During the developing process, we did that by optimizing the response over three different tissue types. The results obtained with this approach were analogous to the ones obtained by the single tissue approach, but the computation time increased by a factor of 3. Since the contrast uniformity is a by‐product of the single‐tissue design approach, we decided to adopt this strategy. Furthermore, the amplitude sweep of each channel is rather mild, in contrast to the pulse trains obtained in 15. This should have a positive effect regarding the robustness to $B_{1}^{+}$ maps inaccuracies, as already anticipated in 6. As the scanner experiments were successful, we believe that the optimal control pulse trains are robust to $B_{1}^{+}$ mapping errors. A thorough stability analysis of the optimal control method could be the subject of further research.

We would like to point out that the procedure outlined in this article might find a local minimum of the design problem. Given the improvement in the objective, and thus in the signal homogeneity, we have shown that even a local minimum solution has attractive properties.

The approach we followed for sequence design differs from the majority of existing methods such as spokes 20, multidimensional RF pulse design 16, 17, 18, 3D spiral nonselective RF pulses 24 and *k*_T_ points 21, 22, 23 which consider the flip angle created by each RF pulse separately. TSE sequences with variable flip angles have been approached by designing multidimensional refocusing pulses that maintain the CPMG condition, either scaling them in amplitude 22 or adaptively redesigning for specific target flip angles 23. Our work follows a different approach, using simple hard pulses for refocusing, with optimization focusing on the overall state of the magnetization. This approach could be seen as signal control by dynamic shimming. By avoiding multidimensional pulses, we simplify the resulting pulse sequences and shorten the achievable echo spacing. The results shown in this article indicate that major improvement in the sequence response can be achieved by acting only on the amplitude and phase settings. The approach could however be complementary to approaches employing multidimensional pulses, with the added benefit of relaxing the constraint from the CPMG condition, since pulses are directly designed to result in stable echo amplitudes.

We have shown how to apply optimal control techniques to the EPG formalism. Optimal control of the Bloch equation is a rather developed field and with this work we hope to inspire future synergies between the two domains. One example thereof could be the combination of Bloch‐equation/EPG approaches to jointly design the excitation pulse (by Bloch equation methods) and the resulting dynamic shimming settings (by EPG methods). This combination can be implemented as a unified numerical optimal control problem. We think that the hybrid design approach Bloch equation/EPG can pave the way to new developments in the sequence design field.

In terms of safety, the sequences we have designed are constrained by peak and total RF power. The same formalism can be used to include constraints on global specific absorption rate and local specific absorption rate 35, 36. We expect that the computation time will increase by adding a large amount of local specific absorption rate constraints. Implementation on parallel computing architectures will make online application possible as is the case for multidimensional RF pulse design 37.

In this work, we have focused on the design of tip angles and corresponding phases. This is not a restriction for the algorithmic approach, since other parameters, for instance the echo spacing, *τ*, could be subject to optimization too. The framework includes the possibility to treat also *τ* as a variable and the derivatives with respect to *τ* can be calculated by ASM. This could be exploited to increase the contrast between different tissue species. An investigation of this aspect goes beyond the scope of this article and we leave it for future work.

The recently developed quantitative MR reconstruction technique based on the EPG signal model 38 has been implemented with a derivative‐free based method 39. The insights provided in our work will make it possible to employ more efficient derivative‐based algorithms for the same reconstruction paradigm. In particular, ASM can be applied for the calculation of the derivatives with respect to parameters as $B_{1}^{+}$, *T*_1_, and *T*_2_ in the same way as we have obtained the derivatives with respect to the pulses amplitude and phase. In general, we believe that ASM can pave the way for a broader application of EPG‐based reconstruction techniques in MRI.

## CONCLUSION

The design of multi spin‐echo sequences is cast as a solution of a minimization problem. The functions involved are differentiable and, by application of the ASM, the exact derivatives can be quickly calculated. This allows one to solve the design problem with standard and efficient derivative‐based methods. The flexibility and efficiency of the optimal control framework can be exploited to design new sequences and to optimize the MR exam in a patient‐specific, online fashion.

## IMPLEMENTATION ASPECTS

The transition matrix, *P*, is given by the product

P=(I⊗RE)S

where *S* denotes the shift operator matrix, *I* is the *N* × *N* identity matrix and ⊗ denotes the kronecker tensor product. R and E the rotation and decay matrices as given in Eqs. (3), (4), (5). *S* is the shift operator matrix.

### Derivatives of *P*

We wish to calculate the derivatives of *P* with respect to a given parameter *η* where *η* represents either *α*, $\phi ,\;x_{\ell }$, or $y_{\ell }$. First note that

$$\frac{\partial P}{\partial \eta }=\frac{\partial (I⊗RE)S}{\partial \eta }=I⊗\left(\frac{\partial R(\eta )}{\partial \eta }E\right)S.$$

We focus thus on the derivatives of R. For the standard setup (no pTx) we easily obtain:

(24) $$\frac{\partial R}{\partial \alpha }=\left(\begin{array}{ccc} -\sin(\alpha )/2 & 1/2\exp(2\iota \phi )\sin(\alpha ) & -\iota \exp(\iota \phi )\cos(\alpha )\\ 1/2\exp(-2\iota \phi )\sin(\alpha ) & -\sin(\alpha )/2 & \iota \exp(-\iota \phi )\cos(\alpha )\\ -\iota /2\exp(-\iota \phi )\cos(\alpha ) & \iota /2\exp(\iota \phi )\cos(\alpha ) & -\sin(\alpha ) \end{array}\right)$$

and

(25) $$\frac{\partial R}{\partial \phi }=\left(\begin{array}{ccc} 0 & -2\iota \exp(2\iota \phi )\sin{\left(\alpha /2\right)}^{2} & \exp(\iota \phi )\sin(\alpha )\\ -2\iota \exp(-2\iota \phi )\sin{\left(\alpha /2\right)}^{2} & 0 & \exp(-\iota \phi )\sin(\alpha )\\ -1/2\exp(-\iota \phi )\sin(\alpha ) & -1/2\exp(\iota \phi )\sin(\alpha ) & 0 \end{array}\right).$$

For the pTx configurations, we need $\partial R/\partial x_{\ell }$ and $\partial R/\partial y_{\ell }$. First we set $\theta \equiv \sum_{\ell =1}^{L}B_{\ell }(x_{\ell }+\iota y_{\ell })$. We can then write

α=|θ| and ϕ=∠(θ).

Setting x=ℜ(θ) and y=ℑ(θ) and applying the chain rule, we observe that

(26) $$\frac{\partial R}{\partial x_{\ell }}=\frac{\partial R}{\partial \alpha }\left(\frac{\partial \alpha }{\partial x}\frac{\partial x}{\partial x_{\ell }}+\frac{\partial \alpha }{\partial y}\frac{\partial y}{\partial x_{\ell }}\right)+\frac{\partial R}{\partial \phi }\left(\frac{\partial \phi }{\partial x}\frac{\partial x}{\partial x_{\ell }}+\frac{\partial \phi }{\partial y}\frac{\partial y}{\partial x_{\ell }}\right)$$

and

(27) $$\frac{\partial R}{\partial y_{\ell }}=\frac{\partial R}{\partial \alpha }\left(\frac{\partial \alpha }{\partial x}\frac{\partial x}{\partial y_{\ell }}+\frac{\partial \alpha }{\partial y}\frac{\partial y}{\partial y_{\ell }}\right)+\frac{\partial R}{\partial \phi }\left(\frac{\partial \phi }{\partial x}\frac{\partial x}{\partial y_{\ell }}+\frac{\partial \phi }{\partial y}\frac{\partial y}{\partial y_{\ell }}\right).$$

To find the derivative terms inside the brackets, note that:

$$\alpha =\sqrt{x^{2}+y^{2}}\;\text{and}\;\phi =\arctan\left(\frac{y}{x}\right)$$

(28) $$\Rightarrow \frac{\partial \alpha }{\partial x}=\frac{x}{\alpha },\;\frac{\partial \alpha }{\partial y}=\frac{y}{\alpha },\;\frac{\partial \phi }{\partial x}=-\frac{y}{\alpha^{2}},\;\frac{\partial \phi }{\partial y}=\frac{x}{\alpha^{2}}.$$

Furthermore we have:

(29) $$\frac{\partial x}{\partial x_{\ell }}=ℜ(B_{\ell }),\;\frac{\partial x}{\partial y_{\ell }}=-ℑ(B_{\ell }),\;\frac{\partial y}{\partial x_{\ell }}=ℑ(B_{\ell }),\;\frac{\partial y}{\partial y_{\ell }}=ℜ(B_{\ell }).$$

Substituting Eqs. (24), (25), (28), and (29) into Eqs. (26) and (27) we obtain the desired derivatives.
